# Serum Hepatocyte Growth Factor Is Associated with Small Vessel Disease in Alzheimer’s Dementia

**DOI:** 10.3389/fnagi.2018.00008

**Published:** 2018-01-23

**Authors:** Yanan Zhu, Saima Hilal, Yuek L. Chai, M. K. Ikram, Narayanaswamy Venketasubramanian, Christopher P. Chen, Mitchell K. P. Lai

**Affiliations:** ^1^Department of Pharmacology, Yong Loo Lin School of Medicine, National University of Singapore, Singapore, Singapore; ^2^Memory Aging and Cognition Centre, National University Health System, Singapore, Singapore; ^3^Departments of Radiology and Epidemiology, Erasmus University Medical Center, Rotterdam, Netherlands; ^4^Departments of Neurology and Epidemiology, Erasmus University Medical Center, Rotterdam, Netherlands; ^5^Raffles Neuroscience Centre, Raffles Hospital, Singapore, Singapore

**Keywords:** hepatocyte growth factor, Alzheimer’s disease, cognitive impaired no dementia, CeVD, small vessel disease

## Abstract

**Background**: While hepatocyte growth factor (HGF) is known to exert cell growth, migration and morphogenic effects in various organs, recent studies suggest that HGF may also play a role in synaptic maintenance and cerebrovascular integrity. Although increased levels of HGF have been reported in brain and cerebrospinal fluid (CSF) samples of patients with Alzheimer’s disease (AD), it is unclear whether peripheral HGF may be associated with cerebrovascular disease (CeVD) and dementia. In this study, we examined the association of baseline serum HGF with neuroimaging markers of CeVD in a cohort of pre-dementia (cognitive impaired no dementia, CIND) and AD patients.

**Methods**: Serum samples from aged, Non-cognitively impaired (NCI) controls, CIND and AD subjects were measured for HGF levels. CeVD (cortical infarcts, microinfarcts, lacunes, white matter hyperintensities (WMH) and microbleeds) were assessed by magnetic resonance imaging (MRI).

**Results**: After controlling for covariates, higher levels of HGF were associated with both CIND and AD. Among the different CeVD MRI markers in CIND and AD, only small vessel disease, but not large vessel disease markers were associated with higher HGF levels.

**Conclusion**: Serum HGF may be a useful peripheral biomarker for small vessel disease in subjects with cognitive impairment and AD.

## Introduction

Alzheimer’s disease (AD) is the most common form of neurodegenerative dementia and a significant source of caregiver morbidity and healthcare burden. The neuropathological hallmarks of AD consist of extracellular deposits of β-amyloid-containing neuritic plaques, as well as intracellular neurofibrillary tangles derived from aggregated, abnormally hyperphosphorylated tau proteins. Besides the aforementioned plaques and tangles, AD is also characterized by synaptic loss (Selkoe, 2002), dysregulated neuroinflammation (Wyss-Coray and Rogers, 2012) and neuronal degeneration (Scheltens et al., 2016). Additionally, cerebrovascular disease (CeVD) has been suggested to play a pathogenic role in dementias including AD (Kling et al., 2013). Several neuroimaging markers of CeVD, such as cortical infarct, lacunes and especially white matter hyperintensities (WMH) are associated with AD and may contribute to disease deterioration (Jellinger, 2002; Moghekar et al., 2012; Toledo et al., 2013; Attems and Jellinger, 2014). Given the prolonged prodromal stages where potential disease-modifying therapies may more likely be efficacious (Jack et al., 2013), the availability of reliable, easily accessible diagnostic and prognostic biomarkers is essential for optimal clinical management, and will also help advance and assess new therapeutic strategies for AD. While cerebrospinal fluid (CSF) and positron emission tomography (PET)-based biomarkers are available for AD pathology (β-amyloid and tau; Villemagne et al., 2017; Zetterberg, 2017) which may be associated with small vessel CeVD (Kester et al., 2014), these are relatively costly and invasive, and research efforts to uncover blood-based biomarkers are ongoing (Henriksen et al., 2014; Zetterberg, 2017).

Hepatocyte growth factor (HGF, also known as scatter factor), originally isolated from liver and known to play a role in liver regeneration (Nakamura et al., 1984), has recently been shown to also regulate various brain functions, including axon outgrowth, neuronal survival, synaptic function and plasticity (Ebens et al., 1996; Nakamura and Mizuno, 2010; Wright and Harding, 2015). While previous studies reported increased HGF levels in the brain and CSF of patients with AD (Fenton et al., 1998; Tsuboi et al., 2003), it is unclear whether peripheral HGF may be related to cognitive impairment and dementia. Furthermore, HGF can regulate vascular functions including angiogenesis (Xin et al., 2001; Morishita et al., 2002), and has also been shown to reduce ischemia-associated infarct volume (Tsuzuki et al., 2001; Shimamura et al., 2004), perhaps by preventing delayed neuronal death (Miyazawa et al., 1998). However, no study has yet examined the association of serum HGF in patients with prodromal dementia (cognitive impairment no dementia, CIND), AD, or concomitant CeVD. Hence, in the present study we examined the association of serum HGF levels with MRI markers of CeVD and cognitive impairment/AD in a memory clinic population.

## Materials and Methods

### Study Cohort

The selection and assessment of the cohort for this case-control study have previously been described (Hilal et al., 2015; Chai et al., 2016a; Zhu et al., 2017). Briefly, patients with subjective complaints of memory loss were recruited from memory clinics at Singapore’s National University Hospital and Saint Luke’s Hospital sites between August 2010 and May 2014. This study was carried out in accordance with the recommendations of Singapore National Healthcare Group Domain-Specific Review Board (NHG-DSRB) guidelines with written informed consent from all subjects. All subjects gave written informed consent in accordance with the Declaration of Helsinki. The protocol was approved by NHG-DSRB (reference: 2010/00017; study protocol number: DEM4233). Clinical, physical, neuropsychological assessments and neuroimaging were performed at the National University of Singapore. In the current study, the research focus is on CIND/AD with concomitant CeVD. Therefore, relevant demographic and medical covariates, including cardiovascular risk factors (see “Materials and Methods” and “Covariates” sections below), and exclusion factors such as previous head trauma, pure vascular dementia (Román et al., 1993), psychiatric illnesses, thyroid disease and non-AD neurodegenerative conditions (e.g., Parkinson’s disease) were collected by administering detailed questionnaire, clinical assessments and review of medical records.

### Covariates

Medical histories of vascular risk factors such as hypertension, hyperlipidemia, diabetes, smoking and cardiovascular disease were collected and classified as absent or present, along with demographic information. Hypertension was defined as systolic blood pressure ≥140 mmHg and/or diastolic blood pressure ≥90 mmHg, or use of antihypertensive medications. Hyperlipidemia is defined as total cholesterol levels of ≥4.14 mM, or on antihyperlipidemic medication. Diabetes mellitus was defined as glycated hemoglobin (HbA1c) of ≥ 6.5%, or on medication. Cardiovascular disease was classified as a previous history of atrial fibrillation, congestive heart failure and/or myocardial infarction. Apolipoprotein E (APOE) genotyping using DNA extracted from the buffy coat of blood samples were as previously described (Chai et al., 2016b) for the determination of APOE ε4 carrier status, defined by the presence of at least one APOE ε4 allele.

### Cognitive Assessments

All subjects underwent cognitive assessments including a comprehensive neuropsychological test battery consisting of executive function, attention, language, visuomotor speed, visuoconstruction, verbal memory and visual memory domains (Hilal et al., 2015), along with standard brief cognitive tests (Mini-Mental State Examination and Montreal Cognitive Assessment, Dong et al., 2012). Diagnoses of cognitive impairment and dementia were made at regular consensus meetings of study clinicians and neuropsychologists, where CIND cases were defined as not meeting DSM-IV diagnostic criteria for dementia (American Psychiatric Association, 1994) but showing impairment in at least one domain of the neuropsychological battery by education-adjusted scores ≥1.5 standard deviations below normal established means in > half of the tests for that domain. AD cases were diagnosed using the National Institute of Neurological and Communicative Disorders and Stroke and the AD and Related Disorders Association (NINCDS-ADRDA) criteria (McKhann et al., 1984). Non-cognitively impaired (NCI) controls were defined as those with subjective memory complaints, but who were cognitively normal on objective neuropsychological assessment.

### CeVD Neuroimaging

Magnetic resonance imaging (MRI) scans were obtained using 3T Siemens Magnetom Trio Tim scanner with a 32-channel head coil at the Clinical Imaging Research Center of the National University of Singapore. Subjects who had claustrophobia, contraindications for MRI, or were unable to tolerate the procedure were excluded from the present analysis. Cortical and lacunar infarcts were graded on T1- and T2- weighted images with Fluid Attenuated Inversion Recovery (FLAIR) sequences. Cortical infarcts were identified as focal lesions involving cortical gray matter with low signal on T1-weighted image and FLAIR, a high signal on T2-weighted image, and hyperintense rim on FLAIR images and center following CSF intensity. This is further aided by tissue loss of variable magnitude, with prominent adjacent sulci and ipsilateral ventricular enlargement. Lacunar infarcts were defined as lesions involving the subcortical regions, 3–15 mm in diameter, with low signal on T1-weighted image and FLAIR, a high signal on T2-weighted image, and a hyperintense rim with a center following CSF intensity on FLAIR. Cortical microinfarcts (CMIs) were graded on T1, T2-weighted and FLAIR sequences, and were defined as hypointense lesions on T1-weighted images, <5 mm in diameter, restricted to the cortex and perpendicular to the cortical surface (Hilal et al., 2016, 2017). WMH grading was based on the Age-Related White Matter Changes (ARWMC) scale Total score (range from 0: absent to 30: diffuse involvement) in five brain regions, i.e., frontal, parieto-occipital, temporal, cerebellum and basal ganglia (Wahlund et al., 2001). Cerebral microbleeds were defined as focal, rounded lesions of hypointensity graded on Susceptibility Weighted Images (SWI) using the Brain Observer Microbleed Scale (BOMBS; Cordonnier et al., 2009).

### Serum HGF Measurement

Non-fasting blood was drawn from study participants into serum-separating tubes, then centrifuged at 2000 *g* for 10 min at 4°C. Serum samples were then mixed well, aliquoted and stored at −80°C for future use. All samples were subjected to only one freeze-and-thaw cycle. Concentrations of HGF were measured in duplicates by a bead-based multiplexing immunoassay technology, MILLIPLEX™ MAP Kit (Millipore Corp, Billerica, MA, USA) as per manufacturer’s instructions. The detectable concentration range of HGF is from 4.0 pg/mL to 15,000 pg/mL.

### Statistical Analyses

Statistical analyses were performed using SPSS software (version 21, IBM Inc., Armonk, NY, USA). For group comparisons, one-way analysis of variance (ANOVA) with Bonferroni *post hoc* tests were used for normally distributed continuous variables (age); Chi-square tests for categorical variables (gender, hypertension, APOE ε4 carrier, hypertension, hyperlipidemia, diabetes, smoking and cardiovascular disease); while non-parametric Kruskal-Wallis ANOVA with Dunn’s *post hoc* tests were used for skewed distributed continuous variable (HGF levels). For regression analyses, HGF levels were logarithmically transformed to ensure normal distribution. In order to assess the association between HGF levels and cognitively impaired groups (CIND and AD), binary logistic regressions with odds ratios (ORs) and 95% confidence intervals (CI) were computed. All the models were initially adjusted for age, and gender, and subsequently for APOE ε4 carrier, hypertension, diabetes and cardiovascular diseases. In order to identify associations between HGF levels and CeVD markers in CIND/AD, we further constructed Poisson regressions models for counts of cortical and lacunar infarcts, cortical microinfarcts and microbleeds with measure of associations as rate ratios (RR) and 95% CI. The interpretation for RR data are as follows: a person with a 10-fold increase in HGF (1 unit of 10-log increment) will be RR times more likely to have 1 additional CeVD count on MRI compared with a person with a lower HGF level. For WMH grading, the visual scores were treated as continuous variables and linear regression models were performed with measures of associations as mean differences with 95% CI. The interpretation of the effect estimates on WMH was as follows: 1 unit of 10-log increment in HGF would give rise to (mean difference) units increase in WMH burden score (ARWMC Total score, see Table 3). All the models were adjusted in a similar fashion as described above. *P* values <0.05 were considered statistically significant.

**Table 1:** Baseline demographics and biomarker levels of cases and controls (*n* = 310).

Characteristics	NCI	CIND	AD
	*n* = 79	*n* = 138	*n* = 93	*P* value
Age, mean (SD)	68.3 (6.0)	71.3 (8.2)	77.4 (7.3)	0.001*
Female, *n* (%)	41 (51.9)	66 (47.8)	59 (63.4)	0.06
APOE ε4 carrier, *n* (%)	14 (17.7)	41 (29.7)	31 (33.3)	0.06
Hypertension, *n* (%)	43 (54.4)	95 (68.8)	78 (83.9)	**0.001^†^**
Diabetes mellitus, *n* (%)	17 (21.5)	53 (38.4)	41 (44.1)	**0.006^†^**
Cardiovascular disease, *n* (%)	4 (5.1)	23 (16.7)	18 (19.4)	**0.02^†^**
Hyperlipidemia, *n* (%)	53 (67.1)	105 (76.1)	67 (72.0)	0.36
Smoking, *n* (%)	18 (22.8)	41 (29.7)	27 (29.0)	0.52
HGF, median (IQR), pg/ml	525.5 (453)	599.1 (503)	689.9 (467)	**0.009^‡^**

AD, Alzheimer’s disease; CIND, cognitive impairment no dementia; HGF, hepatocyte growth factor; IQR, interquartile range; NCI, no cognitive impairment; n, number; SD, standard deviation. *Significant one-way ANOVA. Pairwise *P* values with *post hoc* Bonferroni tests are: NCI vs. CIND, *P* = 0.01; NCI vs. AD, *P* < 0.001; CIND vs. AD, *P* < 0.001. ^†^Significant Chi-Square test. ^‡^Significant Kruskal-Wallis ANOVA. Pairwise *P* values with *post hoc* Dunn’s tests are: NCI vs. CIND, *P* > 0.05; NCI vs. AD, *P* = 0.007; CIND vs. AD, *P* > 0.05.

**Table 2:** Association of HGF with CIND and AD.

Hepatocyte growth factor*	CIND *n* = 138 OR (95%CI)^†^	AD *n* = 93 OR (95%CI)^†^
Model I	**3.93 (1.39–11.11)**	**6.44 (1.68–24.65)**
Model II	**3.84 (1.29–11.41)**	**7.02 (1.47–33.62)**

OR, Odds ratio (in *bold font* when statistically significant). Model I: adjusted for age and gender. Model II: adjusted for age, gender, hypertension, diabetes, APOE ɛ4 carrier and cardiovascular disease. *Log transformed. ^†^Interpretation: the odds of having CIND/AD in a person with a 10-fold increase in HGF (1 unit of 10-log increment) will be OR times the odds in a person with a lower HGF level.

**Table 3:** Association of HGF with magnetic resonance imaging (MRI) markers of cerebrovascular disease (CeVD) in CIND and AD.

Hepatocyte growth factor*	Number of cortical infarcts *n* = 27 RR (95%CI)^†^	Number of cortical microinfarcts *n* = 52 RR (95%CI)^†^	Number of lacunes *n* = 74 RR (95%CI)^†^	Number of cerebral microbleeds *n* = 115 RR (95%CI)^†^	WMH ARWMC mean difference (95%CI)^‡^
Model I	2.74 (0.73–10.20)	**3.29 (1.66–6.53)**	**4.66 (2.23–9.74)**	**5.59 (4.12–7.60)**	**3.30 (1.18–5.41)**
Model II	2.30 (0.63–8.47)	**3.13 (1.59–6.14)**	**4.09 (1.95–8.63)**	**5.51 (4.07–7.47)**	**3.10 (0.95–5.25)**

RR, Rate ratio (in bold font when statistically significant). Model I: adjusted for age and gender. Model II: adjusted for age, gender, hypertension, diabetes, APOE ɛ4 carrier and cardiovascular disease. *Log transformed. ^†^Interpretation: a person with a 10-fold increase in HGF (1 unit of 10-log increment) will be RR times more likely to have one additional CeVD count on MRI compared with a person with a lower HGF level. ^‡^Interpretation: a 10-fold increase in HGF (1 unit of 10-log increment) will give rise to (mean difference) units increase in white matter hyperintensity (WMH) burden score (age-related white matter changes, ARWMC total score).

## Results

A total of 383 subjects (95 NCI, 164 CIND, 124 AD) were recruited between August 2010 and May 2014, of whom 66 (16 NCI, 23 CIND and 27 AD) had insufficient blood samples and seven (3 CIND, 4 AD) had no neuroimaging (none, incomplete, or ungradable MRI), thus leaving 310 (79 NCI, 138 CIND and 93 AD) with available data for analyses. Table 1 shows the demographic and disease factors of the study cohort. Compared with NCI, CIND and AD were older, and had higher prevalence of diabetes mellitus, hypertension and cardiovascular disease. Serum HGF concentrations in the cohort ranged from 58.4 pg/mL to 3020.0 pg/mL. As shown in Table 1, levels of HGF were highest in AD and lowest in NCI, with intermediate levels in CIND. The differences reached statistical significance between NCI vs. AD (*P* = 0.007, Kruskal-Wallis ANOVA with *post hoc* Dunn’s tests). Table 2 shows the multivariate analyses of associations between serum HGF levels and cognitive impairment. Higher levels of HGF were significantly associated with both CIND and AD after correction for age, gender, APOE ε4 carrier, hypertension, diabetes, and cardiovascular diseases. Finally, Table 3 demonstrates the associations between HGF and MRI markers of CeVD. Higher levels of HGF were significantly associated with higher counts of lacunes, cortical microinfarcts and microbleeds, as well as more severe WMH. In contrast, there was no association between HGF and cortical infarct counts.

## Discussion

In this study, we reported higher levels of HGF in both CIND and AD, independent of demographic covariates and cardiac risk factors. These findings are in line with previous reports of higher HGF levels in the brain and CSF of patients with AD (Fenton et al., 1998; Tsuboi et al., 2003), although it is at present unclear whether peripheral HGF changes reflect HGF variations in the central nervous system (CNS). Additionally, the increased HGF were associated with CeVD in CIND and AD, but interestingly, these associations were restricted to markers of small vessel (lacunes, cortical microinfarcts, microbleeds, WMH), but not large vessel (cortical infarct), disease. Previous studies have shown that small vessel disease substantially contributes to long term morbidity in AD and constitutes an important therapeutic target (Arvanitakis et al., 2016; van Veluw et al., 2017). As they affect cerebral arterioles, venules as well as capillaries, detection and quantification of small vessel disease burden remain challenging on neuroimaging (Wardlaw et al., 2013), and the potential availability of complementary blood-based markers for small vessel CeVD may thus be a useful tool for long term management. Interestingly, the persistence of these associations even after controlling for cardiovascular risk factors suggest distinct interactions between HGF and the cerebrovasculature. Furthermore, the specificity of associations between HGF and small vessel CeVD suggests an etiological link between HGF and small, but not large, vessel disease, although the precise underlying mechanisms are at present unclear. Besides its beneficial effect on axon outgrowth, neuronal survival, synaptic function and plasticity (Ebens et al., 1996; Nakamura and Mizuno, 2010; Wright and Harding, 2015), HGF has been reported to attenuate cerebral ischemia-induced blood-brain barrier (BBB) disruption and loss of tight junction protein expression, both of which are features of small blood vessels of the brain (Date et al., 2006). Furthermore Knowland et al. (2014) showed that ischemia-induced tight junction breakdown associated with BBB disruption, but only at the level of small venules. The increased levels of HGF seen in SVD-associated cognitive impairment and AD cases in our study may thus indicate adaptive plasticity responses to small-vessel mediated neuronal damage or degeneration. However, follow-up studies are needed to validate HGF correlations with small vessel CeVD in AD, as well as their associations with other biomarkers, for e.g., those of BBB disruption and ischemic damage.

The strengths of this study include: (1) a relatively large cohort; (2) inclusion of covariates such as age, gender, APOE ε4 carrier, hypertension, diabetes and cardiovascular diseases in analyses to reduce confounding; and (3) use of neuroimaging platforms to detect and quantify CeVD. However, this study has several limitations as well. First, whilst our finding of HGF association with CIND suggests that HGF changes may be an early feature of AD, it is not possible to establish a temporal association between HGF changes and the development of cognitive impairment and AD because of the cross-sectional design of the study. Follow-up longitudinal studies can help address this limitation as well as elucidate the prognostic utility of serum HGF in predicting the course of CeVD and cognitive decline. Second, while we specifically focused on HGF’s association with CeVD in CIND and AD, it is important to follow-up with similar association studies on vascular or post-stroke dementias in order to further evaluate the nature of HGF changes in response to CeVD, and hence its diagnostic utility. Third, the NCI control group was relatively younger and has less burden of CeVD compared to case groups which could have resulted in selection bias and residual confounding. Lastly, whilst the dementia cases have been assessed using consensus criteria for clinical AD diagnosis (NINCDS-ADRDA), there is a lack of neuropathological confirmation at present.

In conclusion, the present study suggests that serum HGF has potential clinical utility as a peripheral biomarker of small vessel CeVD in cognitive impairment and AD. However, follow-up longitudinal studies are needed for further validation.

## Author Contributions

YZ, CPC and MKPL: conception and design; YZ and SH: development of methodology; YZ, SH, MKI, NV and CPC: acquisition of data; YZ, YLC and SH: analysis and interpretation of data; YZ, YLC, SH and MKPL: writing of manuscript; CPC and MKPL: study supervision. All authors have read, revised and approved the final version of the manuscript.

## Conflict of Interest Statement

The authors declare that the research was conducted in the absence of any commercial or financial relationships that could be construed as a potential conflict of interest.

## Abbreviations

AD: Alzheimer’s disease
CeVD: cerebrovascular disease
CIND: cognitive impaired no dementia
CNS: central nervous system
HGF: Hepatocyte growth factor
NCI: non-cognitively impaired participants
NINCDS-ADRDA: National Institute of Neurological and Communicative Disorders and Stroke and the AD and Related Disorders Association
OR: odds ratio
RR: risk ratio
WMH: white matter hyperintensity.
